# Functional diversity and trade‐offs in divergent antipredator morphologies in herbivorous insects

**DOI:** 10.1002/ece3.6262

**Published:** 2020-04-30

**Authors:** Tadashi Shinohara, Yasuoki Takami

**Affiliations:** ^1^ Graduate School of Human Development & Environment Kobe University Kobe Japan

**Keywords:** antipredator adaptation, Cassidinae, morphological defense, morphological diversification, predator–prey interaction

## Abstract

Predator–prey interactions may be responsible for enormous morphological diversity in prey species. We performed predation experiments with morphological manipulations (ablation) to investigate the defensive function of dorsal spines and explanate margins in Cassidinae leaf beetles against three types of predators: assassin bugs (stinger), crab spiders (biter), and tree frogs (swallower). There was mixed support for the importance of primary defense mechanisms (i.e., preventing detection or identification). Intact spined prey possessing dorsal spines were more likely to be attacked by assassin bugs and tree frogs, while intact armored prey possessing explanate margins were likely to avoid attack by assassin bugs. In support of the secondary defense mechanisms (i.e., preventing subjugation), dorsal spines had a significant physical defensive function against tree frogs, and explanate margins protected against assassin bugs and crab spiders. Our results suggest a trade‐off between primary and secondary defenses. Dorsal spines improved the secondary defense but weakened the primary defense against tree frogs. We also detected a trade‐off in which dorsal spines and explanate margins improved secondary defenses against mutually exclusive predator types. Adaptation to different predatory regimes and functional trade‐offs may mediate the diversification of external morphological defenses in Cassidinae leaf beetles.

## Graphical abstract

We revealed defensive function of dorsal spines and explanate margins in Cassidinae leaf beetles and detected functional trade‐offs. These results suggest that adaptation to different predatory regimes and functional trade‐offs contribute to the diversification of external morphologies in herbivorous insects.

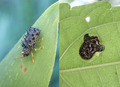

## 1 INTRODUCTION

Enormous morphological diversity within closely related groups of animals can occur by divergent natural selection resulting from predator–prey interactions, which are important determinants of the fitness of prey (Edmunds, 1974; Langerhans, 2007; Stroud & Losos, 2016). Survival is expected to be higher in organisms with any defensive strategies, including morphological defenses, than in those without these defenses. Morphological defenses against predators are divided into two categories based on whether they function before (primary defense) or after (secondary defense) a predator attacks its prey (Edmunds, 1974). Primary defense mechanisms, such as anachoresis, crypsis, aposematism, and mimicry, are aimed at reducing the risk of encounters, detection, identification, and approach by predators via morphological features, coloration, or behavior. Secondary defense mechanisms, such as chemical and morphological defenses, protect prey when attacked by predators. Although these two mechanisms are not mutually exclusive, selective pressures resulting in their evolution may differ. External morphologies can function as both primary (e.g., aposematic spines, Speed & Ruxton, 2005; leaf masquerade, Kuntner, Gregorič, Cheng, & Li, 2016) and secondary (e.g., dorsal spines, Hautier, San Martin, Jansen, Branquart, & Grégoire, 2017; hard armor, Wang, Rajabi, Ghoroubi, Lin, & Gorb, 2018) defenses. Primary defense mechanisms are expected to evolve when it is difficult for prey to survive an attack, whereas secondary defense mechanisms are expected to evolve to facilitate prey escape or survival after an initial attack.

Some organisms adopt a combination of both primary and secondary defenses by single defensive trait (e.g., trash package of green lacewing larvae, Nakahira & Arakawa, 2006). However, there may be a functional trade‐off, in which both strategies cannot be optimized simultaneously. For example, a large body size can physically protect against predation by invertebrates (Whitman & Vincent, 2008), while large prey is likely to be attacked by vertebrates (Karpestam, Merilaita, & Forsman, 2014; Remmel & Tammaru, 2009). Such a trade‐off may also arise between different types of defensive traits within primary or secondary defense mechanisms. Additionally, environmental heterogeneity, including variation in the types or compositions of predators, can exert qualitatively and quantitatively different evolutionary pressure on prey (Brodie, Formanowicz, & Brodie, 1991). The combination of a functional trade‐off between the two strategies and heterogeneity in the predatory environment may generate divergent natural selection for morphological defenses in prey (DeWitt, Robinson, & Wilson, 2000; Langerhans, 2009; Mikolajewski, Johansson, Wohlfahrt, & Stoks, 2006). As a result, environment‐specific sets of defense mechanisms may be elicited (Boeing, Ramcharan, & Riessen, 2006). Therefore, morphological defenses are expected to diversify in response to variation in predators among habitats.

In insects, predation has been invoked as an important selective force influencing clade diversification and morphological divergence across many taxa, such as dragonflies (Hovmöller & Johansson, 2004), flea beetles (Ge et al., 2011), and ants (Blanchard & Moreau, 2017). In particular, in herbivorous insects, predator encounters on host plants may be the major agent of the evolution and divergence of morphological defenses (e.g., Nosil & Crespi, 2006). Adults of Cassidinae (Insecta, Coleoptera, Chrysomelidae) leaf beetles exhibit various external morphologies, such as spines on the dorsal surface of the body and explanate margins surrounding the body, which are hypothesized defensive traits (Chaboo, 2007; Deroe & Pasteels, 1982; Jolivet & Verma, 2002; Olmstead, 1996; Shinohara & Takami, 2017). Species with spines or explanate margins are expected to encounter predators more frequently than species without such morphologies because the former are more likely to use an open habitat (perching on leaf surfaces; Shinohara et al., in revision). Additionally, species with spines or explanate margins tend to utilize different lineages of host plants (monocots and dicots, respectively; Shinohara et al., unpublished data. Thus, they are exposed to different environments, probably including different predators. In addition, Shinohara and Takami (2017) have shown that a Cassidinae species with long dorsal spines is not hunted by the digger wasp *Cerceris albofasciata*, a specialist predator occurring around the wasp's nests, indicating that the spines may contribute to defense against the specialist predator. However, the functions of diverse morphological defenses are largely unclear in Cassidinae, especially the functions against generalist predators found on their host plants.

In this study, we evaluated the defensive function of external morphologies in Cassidinae leaf beetles. To determine whether dorsal spines and explanate margins have different defensive functions against different types of predators, we manipulated the external morphologies of two prey species and presented them to three predator species. We focused on three generalist predator types: a stinger, a biter, and a swallower. If primary defense by crypsis or aposematism plays a role, prey with dorsal spines and explanate margins, indicators of inconspicuousness or unprofitability, are expected to be less frequently attacked by predators than those without these morphologies. This prediction depends on the ability of predators to perceive these indicators from a distance (e.g., visually). This assumption is generally met for the predators evaluated in this study. Primary defenses reduce per capita mortality more substantially than secondary defenses and therefore are under stronger natural selection (Ruxton, Allen, Sherratt, & Speed, 2018). However, adaptation by predators may overcome primary defenses (Ruxton et al., 2018). In this case, secondary defense mechanisms are expected to be elicited. If secondary defense mechanisms play a role, prey with dorsal spines and explanate margins are expected to survive attacks more frequently than prey without these morphologies. We test these hypotheses and discuss the functions of external morphologies and the diversification of antipredator morphologies in herbivores.

## 2 MATERIALS AND METHODS

### 2.1 Prey and predator species

**Figure 1:**
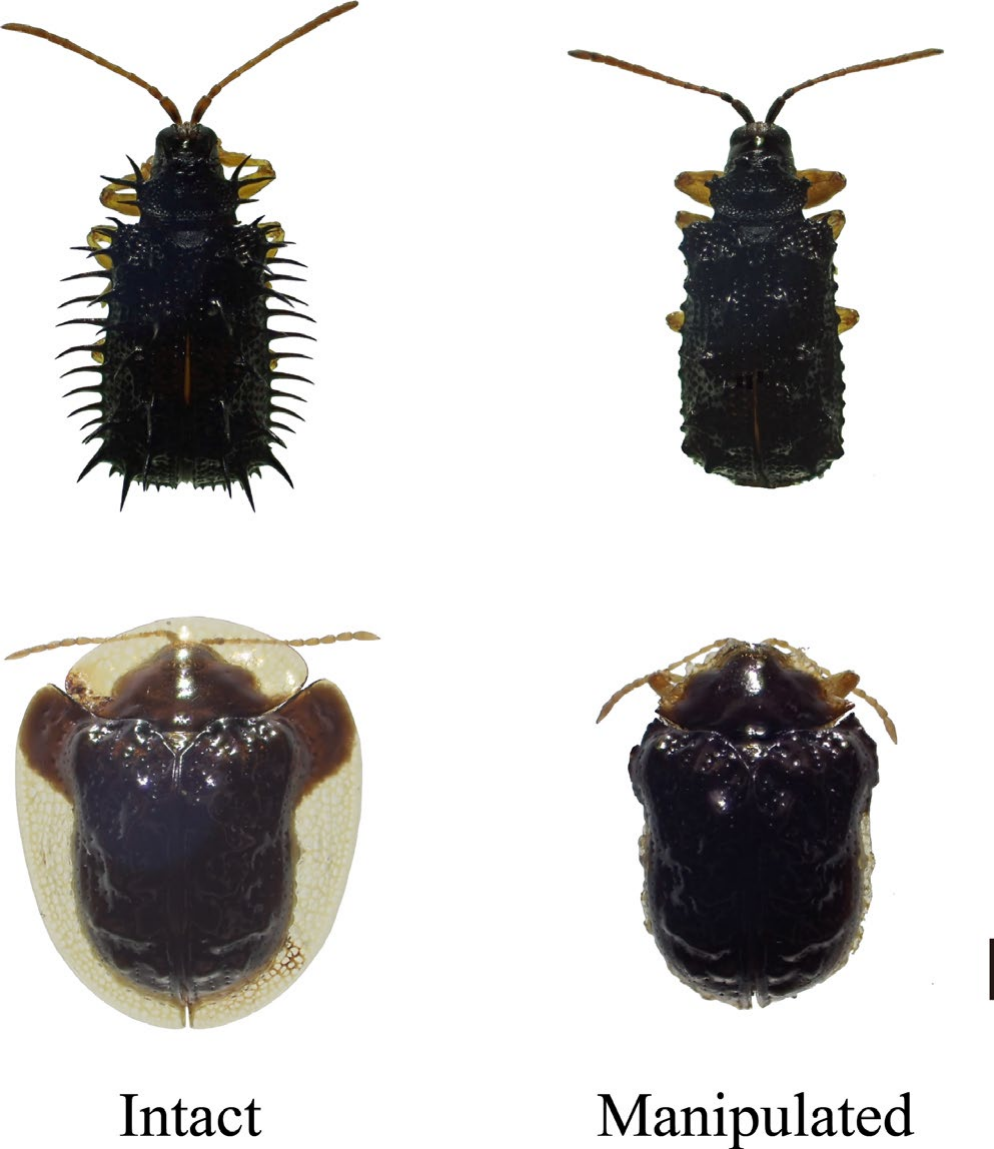
Dorsal aspects of spined prey (*Dactylispa issikii*, upper) and armored prey (*Cassida sigillata*, lower) used in predation experiments. Scale bar, 1 mm

Adults of *Dactylispa issikii* (hereafter, spined prey) and *Cassida sigillata* (hereafter, armored prey), belonging to the subfamily Cassidinae (Insecta, Coleoptera, Chrysomelidae), were used as prey; these species utilize monocot (Poaceae, *Pleioblastus* spp.) and dicot (Lamiaceae, *Isodon* spp.) hosts and have spines and explanate margins, respectively (Figure 1). Because these two host plants do not co‐occur, the habitats of these two beetles are spatially separated. Body lengths of the spined and armored prey were about 5–5.5 mm (from the tip of the head to the end of the elytra) and 6–6.5 mm (from the anterior margin of the pronotum to the end of the elytra), respectively. Explanate margins of the armored prey mainly extend in the lateral directions of the body and allow tight adherence to the flat substrate without a gap, enabling the armored prey to hide their legs and antennae. Many dorsal spines of the spined prey extend in the vertical and lateral directions from the body, and legs and antennae cannot be hidden under spines. These spines are stiff and not bendable. Prey at the teneral stage with immature exoskeletons were excluded from the experiments. Chemical defenses against predators are unlikely in these two prey species because defensive glands have not been found in Cassidinae (Deroe & Pasteel, 1982). Vasconcellos‐Neto (1988) hypothesized that the New World armored species *Chelymorpha cribraria* secretes toxic compounds transported by the hemolymph through the elytral micropunctuation structure, and these compounds are unpalatable to spiders and some birds. However, prey species in this study did not show reflex bleeding or any sign of glandular secretion. The prey species were collected from several sites in Kyoto and Hyogo prefectures, Japan. Prey were reared with their host plants in an incubator at 20°C and used within a month.

Three predator types with distinct feeding habits were used: a stinger (the assassin bug *Sphedanolestes impressicollis*), a biter (the crab spider *Xysticus croceus*), and a swallower (the tree frog *Hyla japonica*). Body lengths of the assassin bug, crab spider, and tree frog were about 13–15 mm (from the tip of the head to the end of the abdomen), 6–9 mm (from the tip of the head to the end of the abdomen), and 25–35 mm (from snout to vent), respectively. The rostrum of the assassin bug was longer than the spine length and explanate margin width, indicating that it can reach the beetle body. Sizes of the chelicerae (the entire appendage excluding the fang) of the crab spider were similar to or slightly shorter than the spine length and explanate margin width, making it difficult to bite beetle body parts covered by spines or external margins. The gape size of the tree frog was wider than the body widths of the spined and armored prey. These three predator species emerge mainly from May to June, when adult prey species are active. The assassin bug and crab spider were found around the host plants of the prey species. The tree frog is an active predator and expected to access the host plants. Preliminary experiments showed that these three predators attacked Cassidinae leaf beetles under laboratory conditions. These three predators were collected from several sites in Kyoto, Osaka, and Hyogo prefectures, Japan. The predators were reared under the same conditions used to rear the prey and were fed whole or cut mealworms several times per week.

To locate prey, the crab spider uses visual cues (Defrize, Lazzari, Warrant, & Casas, 2011) and may also use vibratory cues, similar to the web‐building spider (Wu & Elias, 2014). The tree frog uses visual cues (Freed, 1988). The assassin bug uses visual (Sahayaraj, Martin, Selvaraj, & Raju, 2006; but see Jackson, Salm, & Nelson, 2010) and olfactory cues (Claver ＆ Ambrose, 2001). Similar to frogs, the visual systems of assassin bugs and crab spiders are sensitive to broad ranges of wavelengths (Defrize et al., 2011; Reisenman & Lazzari, 2006). We have previously found that the assassin bug and crab spider prey on a cerambycid beetle and a coccinellid beetle during the daytime, respectively. Thus, the predators in our experiments are able to detect and identify the beetle prey by visual cues. However, the use of substrate‐borne vibrations by crab spiders may be limited in our experimental seting. It is unclear whether prey species are diurnal or nocturnal, but they are found on the host plant in the daytime and thus can encounter the predators.

### 2.2 Predation Experiment

To evaluate the primary and secondary defense mechanisms in Cassidinae leaf beetles, predation experiments were performed using individuals with morphological modifications (Figure 1). The dorsal spines of the spined prey were removed using fine‐tipped tweezers (*N* = 113), and the elytral and pronotal margins of the armored prey were cut off using a scalpel (*N* = 114). This ablation made a slight gap between the substrate and elytra in the armored prey. These manipulations were performed under anesthetization by carbon dioxide gas. Intact controls were also anesthetized and sham‐operated (*N* = 87 and 100 for spined and armored prey, respectively). The prey were used in the predation experiments after recovery from anesthetization (ca. 10 min).

In the predation experiments, 12 combinations of four prey types (experimental and control for two species) and three predator types under laboratory conditions (20–25°C, under room light) were evaluated (Table 1). One prey was introduced together with one predator in a Petri dish (85 mm in diameter and 15 mm in height, but 40 mm in height for the tree frog) and recorded using a digital video recorder (DMX‐CA100; SANYO) for up to 160 min. Prey and predator positions at the start of experiment were arbitrarily determined. Predators were starved for at least 24 hr before the experiments, whereas prey were not starved. The experiments were started in the daytime and sometimes continued to the nighttime owing to the long latency to attack. At most, four prey and predator pairs were observed at a time, in which combinations of prey and predator were arbitrarily arranged. No obstacles were placed between Petri dishes, but they were effectively isolated owing to the round shape and thickness of the glass wall. When no attack occurred in a given experimental period, the pair was observed again for up to nine trials. Petri dishes were cleaned between trials. Experiments were performed from May to October in 2015 and 2016.

**TABLE 1:** Number of trials in the predation experiments. Spined and armored prey are *Dactylispa issiki* and *Cassida sigillata*, respectively (Figure 1). Total and attacked correspond to sample sizes in the tests of primary and secondary defense mechanisms, respectively

Predator	Prey	Treatment	Total (*N*)	Attacked (*N*)
Assassin bug (stinger)	Spined	Intact	21	20 (95.2%)
		Manipulated	28	20 (71.4%)
	Armored	Intact	23	20 (87.0%)
		Manipulated	24	21 (87.5%)
Crab spider (biter)	Spined	Intact	46	21 (45.7%)
		Manipulated	64	20 (31.3%)
	Armored	Intact	45	21 (46.7%)
		Manipulated	31	20 (64.5%)
Tree frog (swallower)	Spined	Intact	38	21 (55.3%)
		Manipulated	44	20 (45.5%)
	Armored	Intact	79	23 (29.1%)
		Manipulated	129	22 (17.1%)

Prior to hypothesis testing, we checked whether the ablation itself affected prey activity by comparing the mean locomotion speed between intact and manipulated prey. Five prey from 12 experimental combinations were randomly chosen (i.e., a total of 60 prey), and the mean speed over 5 min was determined using UMATracker (http://ymnk13.github.io/UMATracker/; Yamanaka & Takeuchi, 2018). The activity of intact spined prey (mean ± standard deviation [*SD*], 10.95 ± 19.99 mm/min, *N* = 15) did not differ significantly from that of manipulated spined prey (10.14 ± 14.97 mm/min, *N* = 15; *t* test, *t* = 0.12, *p* = .90). Activity of intact armored prey (0.54 ± 1.96 mm/min, *N* = 15) did not differ significantly from that of manipulated armored prey (0.47 ± 0.98 mm/min, *N* = 15; *t* test, *t* = 0.13, *p* = .90). Therefore, ablation itself did not influence the activity of prey.

To test the primary defense hypothesis, the efficacies of cryptic or aposematic strategies of prey were evaluated by recording the time from the start of the experiment to the first attack by the predator. An attack was defined as the point at which a predator extended their rostrum to a prey (assassin bug), walked up to a prey and mounted it (crab spider), or opened its mouth and touched the prey (tree frog).

To test the secondary defense hypothesis, the survival of prey after an attack was evaluated. When multiple attacks occurred, survival was only recorded after the first attack. This reflects attacks in the natural environment, in which prey survive after an initial failed attack by escape or strong adhesion to the substrate. However, when a tree frog tried to attack but could not touch a prey, the result of the subsequent attack in which the prey was first touched was recorded. When tree frogs ejected prey from their mouths, prey survival was recorded. When crab spiders bit the dorsal spine of spined prey and then released it, prey survival was also recorded. Some predators were used repeatedly with an interval of at least 1 hr between trials, and if the predator succeeded in predation, the interval was at least 24 hr to control satiety. If this treatment was insufficient to control satiety, the time to attack by the predator is expected to increase as the number of trials involving the predator increases. This was examined below, confirming that there were no effects of repeated testing.

#### 2.2.1 Statistical analysis

The primary defense hypothesis predicts a relatively shorter time to attack by a predator in morphologically manipulated prey than in unmanipulated prey. To test this prediction, the times to the first attack were compared between intact and manipulated prey. Multiple trials were performed with the same pair of prey and predator individuals to increase the sample size of attacks for the examination of the secondary defense mechanism, but only the first trial was used for this analysis to avoid the repeated use of prey. Parametric survival models were constructed using the survreg function of the survival package (Therneau, 2015) in R version 3.3.3 (R Core Team, 2017). The optimal distribution was chosen based on Akaike's information criterion (AIC). The predator ID was included as a random variable to account for the repeated use of predator individuals. Prey that were not attacked within 160 min were treated as censored cases. To examine the effect of experience (e.g., satiety), the number of repeated uses of predators was included as a covariate. Across all six models, this effect was not significant (*p* > .24). Therefore, this covariate was excluded from final models.

The secondary defense hypothesis predicts a relatively lower survival rate in morphologically manipulated prey than in unmanipulated prey. To test this prediction, survival rates were compared between intact and manipulated prey. Generalized linear mixed models (GLMMs; logit link and a binomial distribution) were constructed using prey survival as the response variable. Prey type (intact or manipulated) was included as an explanatory variable, and predator ID was included as a random term. GLMMs were fitted using the glmmML package (Broström, 2017) in R.

## 3 RESULTS

### 3.1 Primary defense mechanism

**Figure 2:**
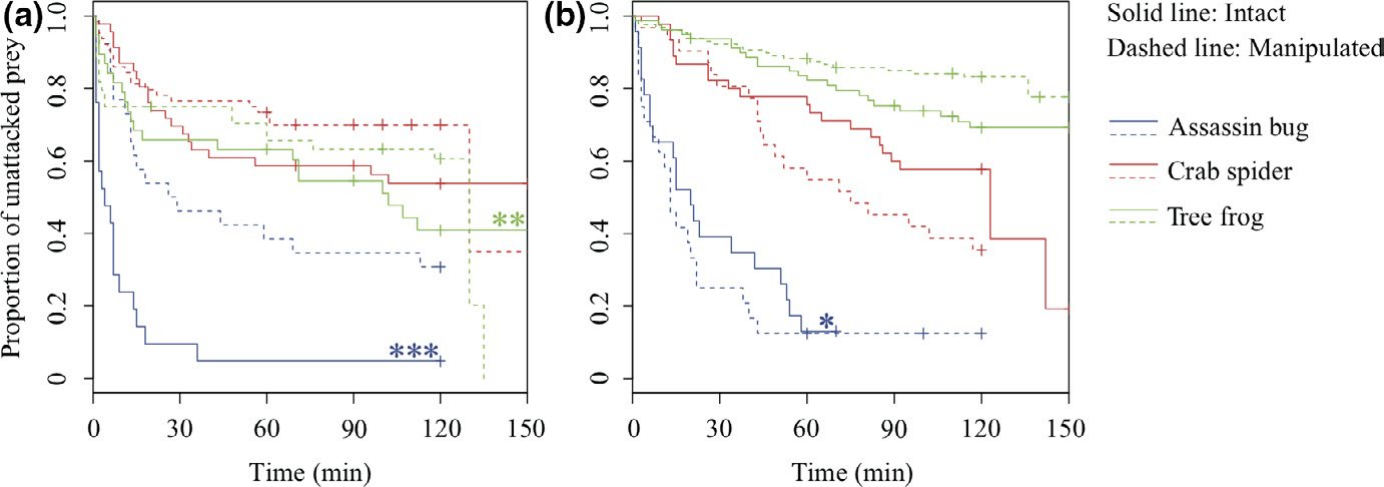
Survival curves of the spined prey *Dactylispa issikii* (a) and the armored prey *Cassida sigillata* (b). Solid line and dashed line indicate intact and manipulated prey, respectively. Blue, red, and green curves indicate the assassin bug (stinger), the crab spider (biter), and the tree frog (swallower), respectively. *p < 0.05; **p < 0.01; ***p < 0.001

Among 241 and 331 trials for spined and armored prey, respectively (Table 1), 200 and 216 were used for the analysis. Contrary to the prediction of the primary defense hypothesis, the times to attack by the assassin bug and the tree frog were significantly shorter for intact spined prey than for manipulated spined prey (Table S1, Figure 2a). Consistent with the prediction, explanate margins reduced the time to attack by the assassin bug (Table S1, Figure 2b).

### 3.2 Secondary defense mechanism

**Figure 3:**
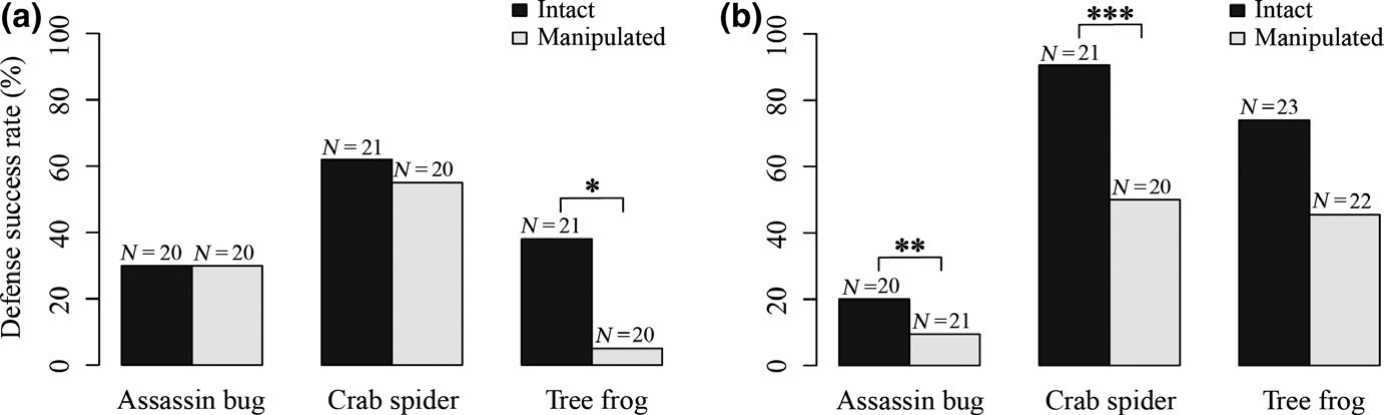
Survival rate of the spined prey *Dactylispa issikii* (a) and the armored prey *Cassida sigillata* (b) against the first attacks by three types of predators. Black and gray bars indicate survival rates of intact and manipulated prey, respectively. The number of attacks is shown above the bars. *p < .05; **p < .01; ***p < .001

We observed attacks by predators in 122 of 241 trials using spined prey (Table 1). Dorsal spines had a significant defensive function against attacks by the tree frog (Table S2, Figure 3a). Eight of 21 intact spined individuals survived, and six of eight individuals who survived were expelled from the mouth after they were initially captured. Expelled prey were weakened but remained alive. By contrast, dorsal spines did not significantly protect against attacks by assassin bugs and crab spiders (Table S2, Figure 3a). Two intact spined individuals were captured by crab spiders who bit their dorsal spines, but these individuals survived.

Attacks by predators were observed in 127 of 331 trials using armored prey (Table 1). Explanate margins had clear defensive functions against attacks by the assassin bug and crab spider (Table S2). Although the survival rate was low after attacks by the assassin bug, the rate was significantly higher for intact armored prey than for manipulated armored prey (Table S2, Figure 3b). Although two assassin bugs tried to suck intact armored prey, they were unable to insert their rostrum under the explanate margins of the prey, and the attacks failed. Intact armored prey survived against attacks by the crab spider at a particularly high rate (90.5%), whereas the survival rate for manipulated armored prey was only 50.0% (Table S2, Figure 3b). Intact armored prey were rarely bitten on their legs or antennae by the crab spider because these body parts were almost completely covered with explanate margins. One intact and one manipulated armored prey survived after they were bitten on their elytral margins by crab spiders. Explanate margins tended to be effective against attacks by tree frogs, although there was only a marginally nonsignificant difference in survival rates between intact and manipulated armored prey (Table S2, Figure 3b). When a tree frog attempted to attack an intact armored prey, it often failed to hold it in its mouth.

## 4 DISCUSSION

Factors contributing to the diversification of external morphologies are a major topic in evolutionary biology. In this study, we examined the functions of two contrasting external morphologies in Cassidinae leaf beetles, dorsal spines and explanate margins, as defensive apparatuses against different types of predators. We hypothesized that the traits evolved as primary defense mechanisms to decrease predator attack rates and as secondary defense mechanisms to increase survival rates after attacks. Our results provided clear evidence for the secondary defense mechanism but mixed support for the primary defense mechanism.

Primary defense mechanisms are mostly ineffective, except for a single prey and predator combination. Attacks by predators were not completely fatal for any combination of manipulated prey and predator (Figure 3), suggesting a relatively low necessity to avoid attacks. Contrary to the prediction, intact spined prey were more likely to be attacked by the assassin bugs and tree frogs than manipulated prey under laboratory conditions. Intact prey are larger than manipulated prey and may be more likely to be detected by particular visual predators. In herbivorous insects, large prey are more likely to be attacked by predators (Karpestam et al., 2014; Remmel & Tammaru, 2009). Alternatively, assassin bugs and tree frogs might avoid manipulated spined prey due to unpalatable chemicals in hemolymph released by ablation (Vasconcellos‐Neto, 1988). By contrast, intact armored prey were more likely to avoid attacks by assassin bugs than were manipulated prey under laboratory conditions. Explanate margins may allow beetles to be more cryptic or unprofitable to the specific predator. For the remaining combinations of prey and predators, we did not detect differences in the time to attack between intact and manipulated prey (Figure 2). The observed differences among prey–predator combinations may depend on the ability of predators to perceive their prey. Prey appearance may differ between experimental and natural conditions. Additionally, our experimental arena may limit the detection and/or identification of prey. To evaluate these defensive morphologies more rigorously, experiments under natural conditions are warranted.

We obtained experimental evidence that the dorsal spines and explanate margins of adult Cassidinae leaf beetles have physical defensive functions against generalist predators. Dorsal spines were found to be effective against attacks by the tree frog. In insects, spines are considered typical defense devices, although their functions have only been revealed in a few taxa (e.g., Honma, Oku, & Nishida, 2006; Hautier et al., 2017; Ito et al., 2016; Mikolajewski & Rolff, 2004; Murphy, Leahy, Williams, & Lill, 2010). Mikolajewski and Rolff (2004) and Ito, Taniguchi, and Billen (2016) showed that the spines of dragonfly nymphs and ant adults protect against gape‐limited predators such as fishes and frogs (swallowers). Thus, recent studies indicate that insect spines themselves are effective against frogs, consistent with our results. However, the relatively small spines of ants do not function as a defensive apparatus against tree frogs (Ito et al., 2016). The lateral spines of a pygmy grasshopper also do not affect survival against frogs and may not function effectively without the aid of other characteristics related to death‐feigning (Honma et al., 2006). Even though the spined prey used in this study were small compared with the gape size of the tree frogs (which are not gape‐limited), six of eight intact prey survived after being expelled from the mouth. This suggests that dorsal spines physically prevent swallowing by tree frogs. Accordingly, these prey are likely to escape from the predator in their natural habitats because natural environments are more complex than experimental conditions, and after being expelled, the prey have the potential to be hidden from the predator. Although we did not detect an effect of dorsal spines against the assassin bug (a stinger) and the crab spider (a biter), Hautier et al. (2017) showed that the spines of coccinellid larva are effective against intraguild coccinellid predators (biters). Murphy et al. (2010) also demonstrated that the spines of limacodid larvae protect against attacks by assassin bugs (stingers) and paper wasps (biters). These differences may be explained by other characteristics of the taxa. The dorsal surface of coccinellid larvae is soft and unprotected; thereby, manipulated larvae are vulnerable to attack. The spines of limacodid larvae can sting enemies, which may be a particularly effective defense.

Explanate margins had a significant defensive function against attacks by the assassin bug (a stinger) and the crab spider (a biter). Additionally, although the difference among prey types was not significant, explanate margins tended to be effective against attacks by the tree frog (swallower). Explanate margins covered the body parts of prey and prevented fatal predatory attacks. In addition to the physical defense by explanate margins, armored prey are able to secure a hold on a substrate in response to a disturbance by wetting their tarsal bristles (Eisner & Aneshansley, 2000). If a beetle clings for sufficiently long, it may be able to thwart a predator's attack. This physical defense combined with improved adhesion may decrease predation rates and/or increase the handling time by predators. Compared with dorsal spines, explanate margins were effective against a wider range of predators, suggesting that explanate margins are more beneficial than dorsal spines in the context of antipredator defense. Recent molecular phylogenetic analyses have indicated that explanate margins have multiple independent origins within Cassidinae, while dorsal spines have a single origin, corroborating the notion that explanate margins are adaptive in the context of predator–prey interactions (Shinohara et al., in revision).

Based on our results, we hypothesize that adaptation to different predatory regimes and trade‐offs between physical defensive functions mediate the diversification of morphological defenses in Cassidinae leaf beetles. Dorsal spines improved secondary defense but weakened primary defense against the swallower, suggesting that there is a trade‐off between the primary and secondary defense mechanisms. Additionally, dorsal spines and explanate margins improved secondary defenses against mutually exclusive predator types; dorsal spines were defensive against the swallower, while explanate margins were defensive against the stinger and biter, suggesting a trade‐off between two defensive morphologies. From an ecological point of view, monocot plants (hosts of most spined Cassidinae beetles) and dicot plants (hosts of most armored Cassidinae beetles) differ in their structure and ecology, implying a difference in predatory regimes; for example, extrafloral nectaries are frequently found in eudicots and attract ants (Weber & Keeler, 2012). The combination of functional trade‐offs and heterogeneous predatory environments may generate divergent natural selection for external morphologies (DeWitt et al., 2000;Langerhans, 2009;Mikolajewski et al., 2006). Divergent natural selection is also suggested by the paucity of species with both spines and explanate margins in Cassidinae (e.g., *Cassidispa* and *Platypria*). These “mixed‐shape” species are only observed as intermediate lineages between those with explanate margins and those with spines (Shinohara et al., in revision), suggesting that carrying both apparatuses is evolutionarily disadvantageous. Interestingly, the trade‐off between physical defensive functions may facilitate the evolution of another primary defense mechanism: cryptic coloration. This phenotype is frequently found in species with explanate margins, the dorsal color of which is often similar to that of the substrate (the leaf of the host plant), and the flattened shape makes them even more concealed in the foliage (Deroe & Pasteels, 1982). In other species, explanate margins are transparent, most likely because invisible armor protects the prey from predators without increasing body size (O’Brien & Kettle, 1979). Evaluations of predator compositions on different host plants as well as the trade‐off between cryptic coloration and physical defense are warranted.

## AUTHOR CONTRIBUTIONS

**Tadashi Shinohara:** Conceptualization (equal); Data curation (equal); Formal analysis (equal); Writing‐original draft (equal). **Yasuoki Takami:** Conceptualization (equal); Formal analysis (equal); Writing‐original draft (equal).

## Supporting information

Table S1

Table S2

## Data Availability

Data have been deposited at the Dryad Digital Repository: https://doi.org/10.5061/dryad.kprr4xh22.
