# Supplementary figures and images for: Functional diversity and trade‐offs in divergent antipredator morphologies in herbivorous insects
Source: Ecol Evol. 2020 Apr 30;10(11):5089–96. doi: 10.1002/ece3.6262 (PMC7297758; doi:10.1002/ece3.6262)

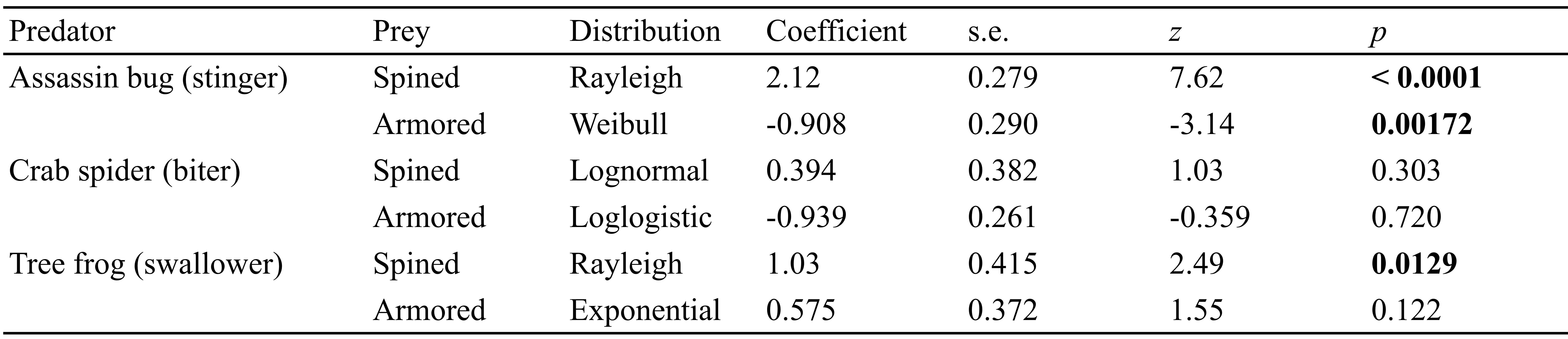

Supplement: Supplementary file 1 — Table S1 [file ECE3-10-5089-s001.jpg]

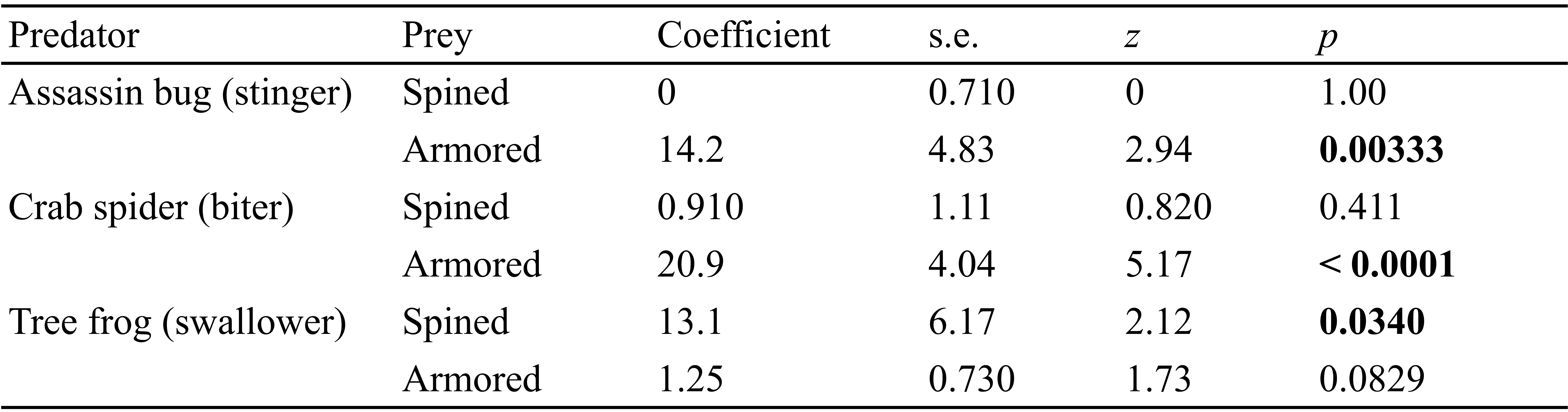

Supplement: Supplementary file 2 — Table S2 [file ECE3-10-5089-s002.jpg]
